# Low level of knowledge about neonatal danger signs and its associated factors among postnatal mothers attending at Woldia general hospital, Ethiopia

**DOI:** 10.1186/s40748-018-0073-5

**Published:** 2018-03-21

**Authors:** Mekdes Mengesha Jemberia, Elsa Tesfa Berhe, Hawi Bersisa Mirkena, Destaw Molla Gishen, Abera Endale Tegegne, Melese Abate Reta

**Affiliations:** 1 Department of Midwifery, Faculty of Health Science, Woldia University, P.O.Box 400, Woldia, Ethiopia; 2 Department of Medical Laboratory Science, Faculty of Health Science, Woldia University, P.O.Box 400, Woldia, Ethiopia

**Keywords:** Knowledge, Neonates, Danger signs, Associated factors, Postnatal mothers, Woldia

## Abstract

**Background:**

Neonatal mortality has persisted high in Ethiopia in spite of many efforts being applied to decrease this adverse trend. Early detection of neonatal illness is an important step towards improving newborn survival. Toward this end, there is a need for the mothers to be able to identify signs in neonates that signify severe illnesses. The aim of this study was to assess knowledge about neonatal danger signs and its associated factors among postnatal mothers attending at Woldia general hospital, Ethiopian.

**Methods:**

Institutional based cross-sectional study design was conducted from January–May, 2017. The hospital that provides antenatal care (ANC), delivery, and postnatal services was purposively sampled. Structured interviewer managed questionnaire was administered to postnatal mothers attending Woldia general hospital. Frequencies, bivariate and multivariate logistic regression were determined using the SPSS software (Version 20).

**Results:**

During the study period 197 mothers attending postnatal care (PNC) service at Woldia general hospital were interviewed. Information on different neonatal danger signs was not provided to 92(46.7%) postnatal mothers during their antenatal clinic attendance by the healthcare providers. The majority of mothers, 174(88.3%) identified less than six neonatal danger signs. The hotness of the body of neonates was the commonly recognized danger sign by 106(53.8%) postnatal mothers. Of the total mothers, 67(34%), 60(30.5%), 56(28.4%), 44(22.3%) recognized unable to breastfeeding, convulsion, lethargy, difficulty in breathing as newly born danger signs, respectively. Out of 197 mothers, 32(16.2%) were giving birth at home. Mother’s age(AOR = 1.33, 95% CI: 1.99–3.08), marital status(AOR = 2.50, 95% CI: 0.29–4.31), mother’s education status(AOR = 3.48, 95% CI:1.57–8.72), husband’s education(AOR = 4.92, 95% CI: 1.29–12.81), attending ANC (AOR = 2.88, 95% CI: 1.15, 4.85), mother’s residence(AOR = 0.78, 95% CI: 0.47–1.65), information about neonatal danger signs(AOR = 3.48, 95% CI 1.40–9.49) had positive association with maternal level of knowledge to identify different neonatal danger signs.

**Conclusion:**

Maternal knowledge level about neonatal danger signs was very low. Therefore, intervention modalities that focus on increasing level of parental education, access to ANC and PNC service are needed.

## Background

The neonatal period is the first 4 weeks of life and represents a vulnerable time in an individual’s life [1]. Early detection of neonatal illness through identifying neonatal danger signs is an important step towards improving newborn survival [2]. Globally, each year about 4 million children die in the first 28 days of life [3], and this accounts for 40% of the death of children under the age of 5 years globally [4]. Most neonatal death occurs in low-income and middle-income countries, particularly in Sub-Saharan Africa [3]. In Ethiopia, about 120,000 babies died at the first 4 weeks of life [5], and this neonatal mortality rate accounts for 42% of under-5 deaths in the country [5, 6]. The majority of this newborn death occurs at home (outside the formal health system) where only a few mothers and families recognize danger signs of newborn illness [3, 4]. It is terrible that many of newborn die every year especially when their death is preventable. The risk of neonatal death is high in the first 24 h of life [5]. The causes of neonatal mortality are not well documented in Ethiopia, however, some studies reported causes such as sepsis, asphyxia, birth injury, tetanus, preterm birth, congenital malformations and unknown causes [5, 6]. Some of the repeatedly reported neonatal danger signs include unable to breastfeed, the movement only when stimulated, low or high temperature, the respiratory rate over 60 breaths per minute, severe chest in drawing and history of convulsion [3]. Recognizing the occurrence of these signs will result in high overall sensitivity and specificity to predict the need for seeking treatment of the newborn [3, 7, 8]. It is estimated that about 75% of neonatal deaths can be avoided [3, 9]. However, this is only possible if mother’s knowledge regarding the neonatal danger signs is good enough to make a decision to seek health care service [3], Because mother’s poor knowledge about newborn danger signs delay cares to seek health care service and treatment [2]. According to a report by Nigatu et al.*,* mothers and husbands higher educational achievement, ANC and PNC attendance and access to television for information were positively associated with mother’s good knowledge about neonatal danger signs [3]. Okawa et al., reported that potential determinants of neonatal danger signs and factors that would delay for sick newborn treatment were categorized into four domains: maternal factors, family factors, antenatal factors, and delivery factors [10].

For the last era, neonatal deaths have gained attention on the global policy agenda because the Millennium Development Goal (MDG) for child survival cannot be met without substantial reductions in neonatal mortality. It is estimated that reduction of under-5 child mortality by two-thirds by 2015, as called for by the MDG, requires a reduction in neonatal mortality of at least 50% [6].

Different tools to facilitate identification of neonatal health problems and management were introduced into the health programs in several countries like Ethiopia. Integrated Management of Newborn and childhood illness developed by world health organization(WHO) was the one which focused on assessment of neonatal danger signs and applies prompt timely treatment [3, 11]. Similarly, Bhutta et al. report, early identification of newborn danger signs by caregivers with prompt and appropriate referral serves as backbone of the programs aiming at reduction in neonatal mortality [12]. According to a report by Yared et al., in Ethiopia, the neonatal mortality rate declined by 1.9% per annum from 1995 to 2010, logarithmically. The early neonatal mortality rate declined by 0.9% per annum and was where 74% of the neonatal deaths occurred [6]. According to 2016 EDHS report all childhood mortality rates have declined over time; the under-5 mortality rate has declined from 116 deaths per 1000 live births 10–14 years prior to the survey (2002–2006) to 67deaths per 1000 live births in the 0–4 years prior to the survey (2012–2016); and the neonatal mortality rate was 29 deaths per 1000 live births [11].

Generally, reducing neonatal morbidity and mortality requires immediate caregiver’s recognition of suggestive danger signs in the neonates and visiting the nearby clinic for early treatment. Trends in Ethiopian society so far recognized mothers as caretakers for the majority of neonates [3, 13]. Therefore, improving maternal knowledge concerning neonatal danger sign is a key entry point [3]. However, studies in the area are limited and inconsistent. Numerous studies have examined the determinants of neonatal mortality, but few have explored maternal levels of knowledge on neonatal danger signs and different danger signs which potentially cause neonatal morbidity [10]. Therefore, the main aim of this study was to explore the maternal level of knowledge on neonatal danger signs and to assess the associated factors among postnatal mothers attending at Woldia general hospital in the study area.

## Methods and materials

### Study area and period

Woldia town is located at 521 km away from the capital city, Addis Ababa. It is found at 2000 m above sea level with a temperature of 22 °C. According to 2007 Ethiopian National population census [11]; the total population of the town is 75,496 people; among them, 38,167 are males and 37,279 are females. According to 2016 Woldia general hospital annual report; the mothers who had gotten PNC service were 2910. The study was conducted from January–May, 2017. The study employed institutional-based cross-sectional design including all postnatal mothers attending at the hospital during the study period. Mentally and physically incapable postnatal mothers to provide a response during data collection period were excluded.

### Study subjects

During the study period, 197 postnatal mothers attending Woldia general hospital for PNC service were interviewed. All eligible postnatal mothers in selected study site were interviewed and neonatal danger signs were assessed through maternal recall.

### Data collection and analysis

Pre-tested and interviewer administered questionnaire adopted from previous studies were employed to record mother’s knowledge about neonatal danger signs, socio-demographic, and economic factors. The questionnaire was translated into the local language(Amharic) to make data collection process simple and translate back to the English language by translators who are perfect(good in English and Amharic language) to check the content validity of the original version. Three trained BSc degree holder health professionals conducted the data collection process.

The total number of correct spontaneous responses to 12 items (neonatal danger signs) with a minimum score of 0 and maximum of 12 was used to measure knowledge of mothers about neonatal danger signs. Accordingly, two categories were developed for neonatal danger sign. Spontaneous response is respondents naming of neonatal danger signs without giving the option of the respected signs. Mothers who mentioned at least six neonatal danger signs were considered as having good knowledge about neonatal danger signs and mothers who mentioned less than six neonatal danger signs were considered as having poor knowledge.

The completeness and consistency of the data were checked, cleaned and double entered to Epidemiological Information (EPI-INFO) software version 3.5.1 and analyzed by Statistical Package for Social Sciences (SPSS) software version 20. Frequencies, proportions, and summary statistics were used to describe the study population in relation to relevant variables and presented by using tables and graphs. A bivariate logistic regression model was fitted to identify factors, which were significant at *p* < 0.2 and then entered into multivariate logistic regression model to identify independent factors those affected mother’s knowledge about neonatal danger signs.

### Operational terms

#### Neonatal danger signs

Are symptoms that complicate the lives of the neonate and happen during the neonatal period.

#### Postnatal care

Care given to a mother for a period of six weeks from the time of delivery using WHO standards contact time within one hour after birth, at 2–3 days, 6–7 days, and extra contacts for those LBW/mothers living with HIV.

#### Knowledge

State of awareness of mothers on neonatal danger signs; defined on the basis of the score (mean).

#### Good knowledgeable

Those mothers who are able to score above the mean of total knowledge based questions.

#### Poor knowledgeable

Those mothers who are able to score below the mean of total knowledge-based questions.

### Ethical considerations

Ethical clearance was obtained from research review and ethical committee of Faculty of Health Science, Woldia University. Communication with the hospital medical director was made through formal letter obtained from Woldia University, Health Science Faculty. After explained the purpose and objective of the study, the researchers obtained written consent of mothers with their age greater than18years. Moreover, written consent was obtained from caretakers on behalf of those with age less than 18 years. Mothers were informed that their participation was on a voluntary basis, and the information obtained from them was kept confidential through no identifier was used.

## Results

### Social-demographic characteristics of the respondents

A total of 197 mothers were interviewed during data collection; of these, about 141(71.6%) are under 18–35 years old with the mean age of 23.9. Mothers who live in urban accounts 120(60.9%), while 77(39.1%) them were living in rural. Of the total interviewed mothers 163 (82.7%) were married, and the majority 125(63.5%) were the housewife and 50(25.4%) of them can’t read and write. About 134 (69.0%) of them are Orthodox Christian followers. Among the total mothers, 106(53.8%) had less than three family size (Table 1).

**Table 1:** Socio-demographic characteristics of mothers attending PNC at Woldia General Hospital, January–May, 2017

Attributes		N	%
Age of mother’s(years)	< 18	22	11.2
	18–35	141	71.6
	36–45	33	16.8
	> 45	1	0.5
Mother’s residence	Urban	120	60.9
	Rural	77	39.1
Mother’s marital status	Single	26	13.2
	Married	163	82.7
	Divorced	6	3.0
	Widowed	2	1.0
Mother’s occupation	Housewife	125	63.5
	Merchant	20	10.2
	Gov’t Employee	31	15.7
	Student	14	7.1
	Others	7	3.6
Family’s monthly income(birr)	< 500	76	38.6
	500–1000	46	23.4
	> 1000	75	38.1
Mother’s educational status	Can’t read and write	50	25.4
	Can read and write	29	14.7
	Grade 1–8	36	18.3
	Grade 9–10	37	18.8
	Grade 11–12	20	10.2
	Diploma & above	25	12.7
Mother’s religion	Orthodox Christian	134	68.0
	Muslim	58	29.4
	Protestant	4	2.0
	Catholic	1	0.5
Mother’s ethnicity	Amhara	165	83.8
	Tigre	19	9.6
	Afar	12	6.1
	Others	1	0.5
Family size	1–3	106	53.8
	4–6	73	37.1
	> 7	18	9.1
Husband’s education status	Can’t read and write	28	14.2
	Can read and write	38	19.3
	Grade 1–8	31	15.7
	Grade 9–10	34	17.3
	Grade 11–12	20	10.2
	Diploma & above	46	23.4
Distance to health institution	< 5 km	86	43.7
	5-20 km	63	32.0
	> 20 km	48	24.4

### Antenatal and postnatal care utilizations of mothers

Among 197 mothers, 172(87.3%) of them had ANC follow-up at health institution; and about 13 (6.6%), 38 (19.3%) and 52(26.4%) had 1st visit, 2nd visit, 3rd visit respectively, and 72(36.5%) of them had completed ANC visit. From total participants, only 122(61.9%) had gotten information about PNC followed. The majority of mothers, 99(50.3%) got information from healthcare providers. Most of the study participants 127(64.0%) don’t know the right postnatal period (PNP) follow-up time. Of the total mothers, 107(54.3%) of them did not know the PNP is a danger time for mothers and neonates. The reason to attend current PNC was 52(26.4%) excessive bleeding, 41(20.8%) neonatal infection, 39(19.8%) unable to breastfeeding. Out of the total respondents, 32(16.2%) delivered their current baby at home (Table 2).

**Table 2:** Antenatal and postnatal care utilization of mothers attending PNC at Woldia General Hospital, January–May, 2017

Attributes		N	%
Do you know ANC follow up time?	Yes	69	35.0
	No	128	65.0
Did you attend ANC?	Yes	172	87.3
	No	25	12.7
How many ANC visit did you attend?	1 Visit	10	5.1
	2 Visit	38	19.3
	3 Visit	52	26.4
	4 Visit	72	36.5
Did you get information about postnatal service?	Yes	122	61.9
	No	75	38.1
What is your source of information about PNC Service?^a^	Health Provider	99	50.3
	Health Extension workers	17	8.6
	Community Conversation	1	0.5
	Media(TV, Radio)	5	2.5
	Traditional Birth Attendant	0	0
Knowing when PNC starts	On the date of delivery (1-6 h)	41	20.8
	6 h-6 days	18	9.1
	6–7 days	5	2.5
	> 7 days	7	3.6
Knowing PNP is danger time for mothers & neonates	Yes	90	45.7
	No	107	54.3
Do you know danger signs of PNP?	Yes	95	48.2
	No	24	12.2
Types of PNP Danger signs for mothers and neonates^a^	Excessive bleeding	100	50.8
	Neonatal infections	64	32.5
	Unable to breastfeed	68	34.5
	Vomiting	61	31.0
	Others	16	8.1
Reasons to attend current PNC service^a^	Excessive bleeding	41	20.8
	Neonate infections	52	26.4
	Unable to breast feed	39	19.8
	Others	66	33.5
What education did you get during PNC attending time? ^a^	Personal hygiene	128	65.0
	Immunization	136	69.0
	Family planning	135	68.5
	Breast feeding	109	55.3
	Neonatal infection	92	46.7
	Care to ill neonates	66	33.5
Did you get the education about your neonate danger signs during PNC?	Yes	105	53.3
	No	92	46.7
What education did you get about your neonate during PNC? ^a^	The advantage of breastfeeding	91	46.2
	Cord care	62	31.5
	Eye care	54	27.4
	Thermoregulation	54	27.4
	Different immunization	63	32.0
	About neonatal danger signs	53	26.9
	Others	4	2.0
Where did you deliver your current baby?	Home	32	16.2
	Hospital	149	75.6
	Health Center	16	8.1
How did you deliver?	Spontaneous vertex delivery	132	67.0
	Cesarean section	54	27.4
	Instrumental	11	5.6

^a^ Multiple responses of respondents

### Sources of information for mothers about neonatal danger signs

**Fig. 1:**
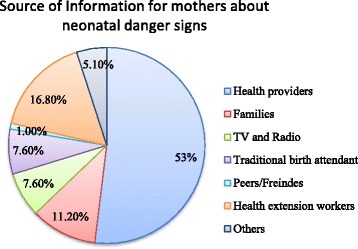
Source of information for mothers on neonatal danger signs

The study revealed that about 105(53%) of mothers attending PNC at Woldia general hospital had gotten information about neonatal danger sign from health providers, while 33(16.8%) of mothers had gotten information from health extension workers (fig. 1).

### Maternal level of knowledge on neonatal danger signs

**Fig. 2:**
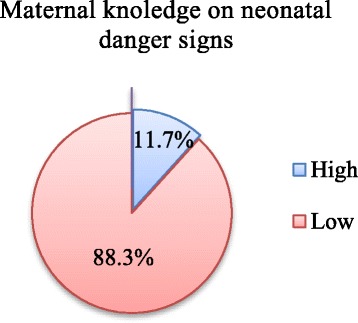
Maternal level of Knowledge on Neonatal danger signs

The scoring of neonatal danger signs was evaluated and scored. For mothers who were able to identify less than six neonatal danger signs were classified as having a low level of knowledge and those who scored more than six were classified as having a good level of knowledge on neonatal danger signs. The majority of mothers, 174(88.3%) had a low level of knowledge. Twenty-three (11.7%) of mothers had good levels of knowledge about neonatal danger sign (95% CI 7.6, 16.3). The most commonly reported source of information was health-care providers (53%) (Fig. 2).

### Mother’s recognition of neonatal danger signs

**Fig. 3:**
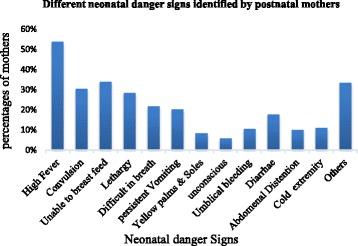
Different neonatal danger signs identified by postnatal mothers

The hotness of the body was the commonly recognized neonatal danger sign by 106(53.8%) postnatal mothers. Out of 197 mothers, 67(34%), 60(30.5%), 56(28.4%), 44(22%), 43(21.8%), 40(20.3%), 35(17.8%), 22(11.2%), 21(10.7%), 20(10.2%), 17(8.6%) identified unable to breastfeeding, convulsion, lethargy, difficulty in breathing, persistent vomiting, diarrhea, coldness, umbilical bleeding, abdominal distention, and yellowness of palms and soles as newborn danger signs, respectively(Fig. 3)**.**

### Factors associated with maternal level of knowledge about neonatal danger signs

After controlling for socio-demographic, economic, and maternal factors; age of mother’s, mother educational status, mothers marital status, Mother’s occupation, husband educational status, attending ANC, Mother’s residence, Distance to health institution and having neonatal danger signs information to mother were the factors that significantly affect maternal levels of knowledge.

Women whose age 18-35 years were 33%(AOR = 1.33, 95% CI: 1.99–3.08) more likely to be knowledgeable as compared to mothers who are < 18 years old. Women who are widowed had two times less likely (AOR = 2.50, 95% CI: 0.29–4.31) to identify at least six neonatal danger signs. Mothers who are diploma and above education level were three times (AOR = 3.48, 95% CI: 1.57–8.72), and more than three times (AOR = 3.05, 95% CI: 1.83–6.90) to be knowledgeable about neonatal danger signs as compared to mothers who can’t read and write, respectively. Similarly, mothers whose husbands are preparatory school education(AOR = 4.92, 95% CI:1.29–12.81) were nearly four times more likely to mention at least six neonatal danger signs as compared to husbands who can’t read and write. Furthermore, mothers who attended ANC during the last pregnancy were two times more likely to head knowledge (AOR = 2.88, 95% CI 1.15, 4.85) about neonatal danger signs as compared to those who did not follow. Mothers who live in the urban area were 22% more likely to have knowledge (AOR = 0.78, 95% CI: 0.47–1.65) about neonatal danger signs as compared to those living in rural area. Likewise, mothers who had got information about danger signs increased their knowledge about neonatal danger signs by 3 times (AOR = 3.48, 95% CI 1.40–9.49) (Table 3).

**Table 3:** Factors associated with mother’s knowledge on neonatal danger signs at Woldia general hospital, January–May, 2017

Characteristics		Knowledge on neonatal danger sign		Crude OR (95% CI)	Adjusted OR (95% CI)
		Good(N)	Poor(N)		
Age of mother’s(year)	< 18	1	21	1	1
	18–35	20	121	1.62(1.41–2.96)*	1.50(0.19–2.41)
	36–45	2	31	3.46(2.30–5.71)*	1.33(1.99–3.08)**
	> 45	0	1	0.84(0.41–3.16)*	0.75(0.57–1.95)
Marital status	Married	19	144	1	1
	Single	3	23	1.80(1.54–6.10)	1.60 (0.39–4.32)
	Divorce	0	6	2.88(0.46–4.31) *	1.33 (0.89–4.08)**
	Widowed	1	1	3.94 (0.31–4.26)*	2.50 (0.29–4.31)**
Mother education	Can’t read and write	1	49	1	1
	Can read and write	4	25	10.86(2.50–75.43)*	4.50 (2.18–7.51)
	Grade 1–8	2	34	9.83(2.61–55.65)*	5.33 (2.99–6.08)
	Grade 9–10	7	30	4.54 (0.31–5.26)	7.72(1.39–63.46)**
	Grade 11–12	2	18	6.18 (2.42–11.51)	3.05 (1.83–6.90)**
	Diploma & above	7	18	10.2 (5.43–23.01)*	3.48 (1.57–8.72)**
Mother’s occupation	Housewife	12	113	1	1
	Merchant	2	18	9.76(3.50–45.23)	4.05 (2.26–8.37)
	Gov’t Employee	6	25	10.83(2.61–95.74)*	5.57 (2.82–9.81)**
	Student	2	12	9.83(1.38–83.27)*	3.48 (0.78–5.89)**
	Others	1	6	3.11 (1.26, 8.48)	1.33 (0.28, 7.18)
Husband’s education	Can’t read and write	1	27	1	1
	Can read and write	3	35	2.52(0.81–4.85)*	1.62(0.91–2.86)
	Grade 1–8	1	30	2.66(1.40–4.81)*	0.96(0.40–1.81)
	Grade 9–10	5	29	2.88(0.37–4.65)	0.78(0.47–1.55)
	Grade 11–12	3	17	9.73 (3.44–27.96)	4.92 (1.29–12.81)**
	Diploma & above	10	36	13.8 (4.12–44.54)*	4.81 (1.43–12.46)
ANC	Yes	22	150	6.71 (4.27–17.49)*	2.88 (1.15, 4.85)**
	No	1	24	1	
Place of delivery	Home	1	31	0.28 (0.23–2.57)	
	Hospital	19	130	2.18 (1.44–3.63)*	
	Health Center	3	13	1	1
Mother’s residence	Urban	19	101	2.77 (1.55, 4.93)*	0.78(0.47–1.65)**
	Rural	4	73	1	1
Distance to health institution	< 5 km	14	72	5.66 (2.82–7.82)	2.72(0.46–3.85)
	5-20 km	7	56	1.67 (0.98–2.49)*	0.46(0.30–0.81)**
	> 20 km	2	46	1.33 (0.88–7.89)*	0.78(0.47–1.75)**
Danger signs information to mother	Yes	20	85	7.77 (3.96–16.07)	3.48 (1.40–9.49)**
	No	3	89	1	1

* P-value < 0.2; ** p-value < 0.05

## Discussion

This institutional based cross-sectional study has assessed the levels of maternal knowledge and the associated factors on neonatal danger signs among mothers attending postnatal care at Woldia general hospital. Reduction of neonatal and infant mortality to an acceptable level is impossible without good maternal knowledge level regarding neonatal danger signs. This is because of the fact that, these danger signs are the entry point to provide comprehensive neonatal health care [3].

In this study, the prevalence of mothers’ good knowledge (mothers who mentioned at least six and above neonatal danger sign) was found to be 11.67%. The finding reported that most mothers have a very poor level of knowledge 174(88.3%) about neonatal danger signs. This is slightly higher than the finding reported from Kenya that 84.5% of the maternal level of knowledge is poor to identify at least three neonatal danger signs from eight mentioned danger signs [8]. Similarly, low maternal levels of knowledge to identify at least one neonatal danger signs was reported from Southwestern Rural Uganda [2]. This may be due to information on danger sign was not adequately disseminated to mothers both during antenatal and PNC period [8]. From total PNC attending women, 74.7% of respondents had formal education from elementary to higher education. Similarly, a study conducted at Addis Ababa reported that mothers attending PNC (35%) have primary school certificate [14]. Maternal education plays a major role in the understanding of neonatal danger signs. In this study mothers who had diploma certificate and above were three times (AOR = 3.48, 95% CI: 1.57–8.72) more likely to be knowledgeable about neonatal danger signs as compared to mothers who can’t read and write. This is in line with neonatal danger signs knowledge level reported from North West of Ethiopia that mothers having secondary and above educational level increased the odds of their knowledge by nearly three times [3]. The possible justification for this could be educated mothers attain knowledge about the neonatal disease and maternal health through their academic life.

The distance of mothers from health institutions can also determine for appropriate ANC and PNC service utilization; our study revealed that 24.4% of mothers travel > 20 km to get PNC service at Woldia general hospital. Mothers who live in the urban area were 22% more likely to have knowledge (AOR = 0.78, 95% CI: 0.47–1.65) about neonatal danger signs as compared to those living in rural area. In contrast to this, the study reported from Southwestern rural Uganda revealed that no association between mothers residence and levels of knowledge on neonatal danger signs [2]. This may be due to those mothers who live far away from health institutions would not have access to ANC and PNC service and would have low access to information about neonatal danger signs.

In our study about 32(16.2%) of women delivered at home (outside the formal health system). In contrast to our report, in India, about 200(70.5%) deliveries take place at home attended by untrained personnel [15]. This difference may be due to study area difference that our study was an institution based whereas Indian study was community-based. Regarding associated factors; maternal knowledge, mother’s age, educational status, marital status, occupation, attending ANC, residence, distance to health institution, danger signs information to the mother, husband educational status were significantly affect maternal levels of knowledge to identify at least six neonatal danger signs in our study. Similarly, the study done in Kenya reported that mother’s education level, PNC accompaniment by Spouse, danger signs information to mother were factors positively associated with better knowledge on neonatal danger sign [8].

## Conclusion

Investigators agreed that mothers can have a great role in caring new born baby and identifying neonatal danger signs. The findings of our study revealed that there is a poor understanding of neonatal danger signs 174 (88.3%). Even participant’s record has the highest number of ANC attendance (87.3%) and institutional deliveries (83.7%), the existing knowledge gap in this key area of neonatal danger signs affect the success of childcare services; this needs to increase educational efforts aimed for all pregnant and delivered women in the hospital as well as in the community.

## Abbreviations

ANC: Antenatal care
AOR: Adjusted odds ratio
CI: Confidence interval
EDHS: Ethiopian Demographic and Health Survey
EPI-INFO: Epidemiological Information software
HIV: Human immunodeficiency virus
LBW: Low birth weight
MDG: Millennium Development Goal
PNC: Postnatal care
PNP: Postnatal period
SPSS: Statistical package for social sciences
WHO: World health organization
